# Seed Watermelon (*Citrullus mucosospermus* (Fursa))-Derived Coniferyl Alcohol as a Functional Ingredient in Remedies for Dry Skin: Evidence of Facilitated Lipogenesis in Human Sebocytes

**DOI:** 10.3390/molecules30163360

**Published:** 2025-08-13

**Authors:** Shingo Fujita, Shoki Inoue, Christos C. Zouboulis, Takashi Fukuda, Toshiharu Hashizume, Tomohiro Itoh

**Affiliations:** 1 Hagihara Farm Co., Ltd., Tawaramoto, Nara 636-0222, Japan; hashizume@suika-net.co.jp; 2 Laboratory for Molecular Chemistry of Aquatic Materials, Department of Life Sciences, Graduate School of Bioresources, Mie University, Tsu 514-8507, Japan; 3 Departments of Dermatology, Venereology, Allergology and Immunology, Staedtisches Klinikum Dessau, Brandenburg Medical School Theodore Fontane and Faculty of Health Sciences, 06847 Dessau, Germany; christos.zouboulis@mhb-fontane.de; 4 Laboratory of Aquatic Food Science, Department of Fisheries, Faculty of Agriculture, Kindai University, Nara 631-8505, Japan; fukudata@nara.kindai.ac.jp

**Keywords:** coniferyl alcohol, seed watermelon, lipogenesis, SZ95 cells, linoleic acid

## Abstract

Sebum secreted by sebaceous glands mixes with sweat to form a protective film that aids in maintaining skin health. Reduced sebum production compromises such barrier functions, potentially leading to severe itchiness and inflammation. Therefore, incorporating moisturizers with ingredients promoting sebum secretion is desirable. Wild watermelon possesses moisturizing and antioxidant properties, and its extracts are utilized in skin cosmetics and supplements. This study investigates whether seed watermelon (*Citrullus mucosospermus* (Fursa))—a species closely related to wild watermelon—influences sebum synthesis and can serve as a skin cosmetic raw ingredient. Several bioactive compounds—including coniferyl alcohol, coniferin, and *p*-coumaryl alcohol—were identified in the active third fraction of the fruit extract. Subsequently, SZ95 sebocytes stimulated with linoleic acid were stained using Oil Red O to detect lipogenesis facilitated by the identified bioactive compounds. Coniferyl alcohol promoted linoleic acid-stimulated lipogenesis by approximately 2.2-fold at a concentration of 300 µM. Lipidomic analyses confirmed an increase in total lipid content following coniferyl alcohol treatment, with notable increases in cholesterol ester, cardiolipin, and simple lipid content. Overall, these findings suggest that seed watermelon contains compounds that do influence sebum synthesis. Consequently, skin cosmetics containing seed watermelon fruit extracts with linoleic acid may benefit individuals with dry skin.

## 1. Introduction

The sebaceous gland is a skin appendage comprising two types of tissues: sebaceous gland lobules and sebaceous gland ducts. Sebocytes differentiated in the basal cell layer synthesize and accumulate sebum in the sebaceous gland lobules. Thereafter, they are destroyed upon apoptosis, and their sebum contents are entirely released into the sebaceous gland ducts (a type of holocrine secretion) [1]. Human sebum is primarily composed of triglycerides and fatty acids (57.7%), wax esters (26%), and squalene (12%) [2]. When secreted from the glands, sebum mixes with sweat released from the apocrine glands and forms a sebum barrier on the skin surface. Notably, exposure to external factors, such as dryness, ultraviolet (UV) rays, chemical substances, and pathogenic microorganisms, poses risks to the skin surface and overall skin health. However, the sebum barrier mitigates the loss of body moisture through evaporation, effects of external physical stimuli and chemical substances, and invasion of pathogenic microorganisms [3,4].

Sebum secretion from sebocytes is regulated by the male hormone testosterone, which is converted to 5α-dihydrotestosterone (DHT) by the enzyme 5α-reductase [5]. DHT, in turn, stimulates the sebaceous glands, promoting sebocyte proliferation and thereby increasing sebum production and secretion [6]. In general, sebum secretion is regulated by various factors such as androgens [6], growth factors [7], neurotransmitters [8], and fatty acids [9,10,11]. Additionally, age influences the amount of sebum secreted from the sebaceous glands in humans. Sebum production peaks between the ages of 17 and 20 years and gradually decreases with age [12]. Decreased sebum synthesis can lead to skin conditions, such as asteatotic eczema, xeroderma, and dry skin, with one in three elderly people being affected by dry skin [13,14]. While symptom management is commonly addressed via the use of medical-grade moisturizers, reactivating diminished sebaceous gland function has also been reported as a viable therapeutic approach [15,16]. For example, the administration of dehydroepiandrosterone has shown efficacy in individuals with significantly reduced sebum production due to menopause or ageing [17,18]. Although topical corticosteroids are frequently prescribed to treat various dermatologic conditions, concerns have been raised regarding their adverse effects under excessive use [19,20]. In some cases, steroid-induced side-effects can exacerbate xeroderma, complicating treatment approaches and increasing the likelihood of recurrence [21]. In this regard, non-steroidal compounds that have been shown to sustainably and fundamentally enhance sebum production and secretion include undecylenic acid and bryonolic acid [22,23]. Previous studies have shown that the topical application of creams containing undecylenic acid promotes the enlargement of sebaceous glands inside hamster ears and improves the content of epidermal lipids on the human forehead [22]. Similarly, treatments with bryonolic acid not only promote the enlargement of sebaceous glands inside hamster ears but also increase squalene levels in their epidermal lipids [23]. Bryonolic acid is an acidic pentacyclic triterpenol compound produced by Cucurbitaceae plants, such as loofah and melon; this suggests that other Cucurbitaceae plant species may also contain compounds that can promote sebum synthesis.

Watermelon (*Citrullus lanatus*), a member of the Cucurbitaceae family, is cultivated worldwide and known for its sweet fruits containing red or yellow flesh. Contrastingly, wild watermelon (*Citrullus lanatus* sp.), believed to have originated from the Kalahari Desert in central South Africa, is characterized by its white, unsweetened flesh [24]. In the Republic of Botswana, of which the Kalahari Desert occupies a large part, the indigenous people store the fruits of wild watermelon as a valuable water resource for the dry season and also utilize them for food and washing their bodies [25]. Wild watermelon can be stored for long periods without spoiling; this suggests that its fruit contains various functional compounds [26,27]. Notably, it is already being used as a raw material in the development of skin supplements and cosmetics. Moreover, it demonstrates remarkable properties, such as disease resistance and heat tolerance, and is used as a breeding material to develop other watermelon cultivars [28,29]. One such cultivar, the seed watermelon (*Citrullus mucosospermus* (Fursa)), is a species that retains many wild-type characteristics. It is primarily consumed in China, where the seed ovules are eaten similarly to nuts. It shares several characteristics with the wild watermelon, such as the appearance, flesh colour, and drought resistance, suggesting that it may also contain natural compounds proving effective for the skin. However, the potential of seed watermelon as a cosmetic ingredient has not been previously investigated, and it has received little attention in the field of cosmetic science. Therefore, in this study, we investigate the possibility of developing skin cosmetics using the seed watermelon fruit as a raw ingredient and also seek to identify bioactive compounds in the fruit that can directly activate sebocytes.

## 2. Results

### 2.1. Isolation of Cinnamyl Alcohol Analogues from Seed Watermelon Fruit

**Figure 1:**
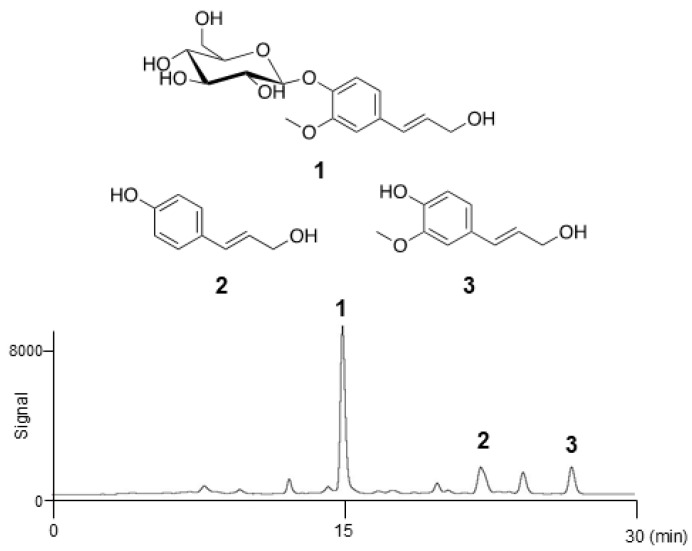
Structures of coniferin (1), *p*-coumaryl alcohol (2), and coniferyl alcohol (3), and the HPLC chromatogram.

The crude extract of seed watermelon fruits was separated using the InertSep C18 column (GL Sciences Inc., Tokyo, Japan), to yield seven fractions (100% H_2_O; 9:1, 4:1, 7:3, 3:2, and 1:1 (*v*/*v*) H_2_O–CH_3_OH; and 100% CH_3_OH). The activity of all fractions for linoleic acid (LA)-stimulated lipogenesis was checked. All fractions showed this activity except for the 100% H_2_O fraction (Figure S1). Because of high yield, the third fraction (56.2 mg) was selected and used for further experiments. This material obtained in 4:1 H_2_O–CH_3_OH, was further purified via reverse-phase high-performance liquid chromatography (HPLC) employing a C-18 packed column. The activity of all peaks in the HPLC chromatogram was checked, and three compounds were confirmed to be active (Figure S2). Coniferin (1), *p*-coumaryl alcohol (2), and coniferyl alcohol (3) were eluted after 15, 22, and 26 min, respectively (Figure 1). The fractions corresponding to the peaks were collected and concentrated to yield colourless powders of pure 1 (3.2 mg), 2 (0.7 mg), and 3 (1.0 mg). All spectral data obtained for these compounds were identical to the commercially available standards or those reported previously, as shown in Figure 1 [30].

### 2.2. Assessment of Cytotoxicity of Isolated Cinnamyl Alcohol Analogues in Terms of Effect on SZ95 Sebocyte Viability

**Figure 2:**
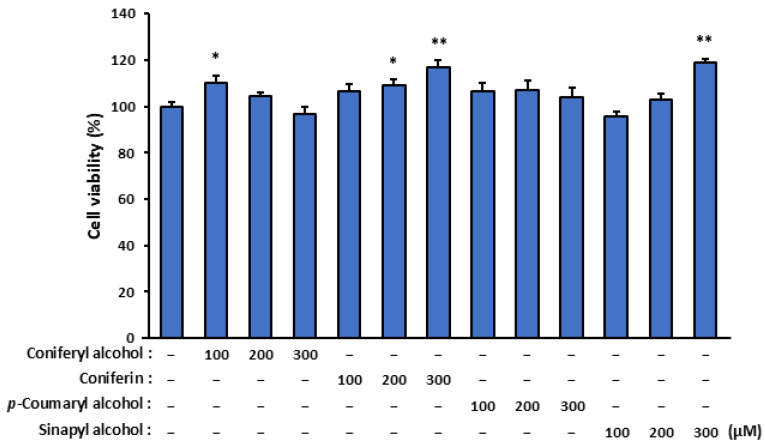
The effect of isolated cinnamyl alcohol analogues on the viability of SZ95 sebocytes. The SZ95 cells were treated with specific concentrations (100, 200, and 300 µM) of the analogues—coniferyl alcohol, coniferin, *p*-coumaryl alcohol, and sinapyl alcohol—for 24 h. Cell viability was assessed using a WST-1 assay. The data are presented as means ± standard error (*n* = 12) and were analyzed for statistically significant differences using Student’s *t*-test, with different letters indicating the differences at * *p* < 0.05 and ** *p* < 0.01 vs. SZ95 cells with no cinnamyl alcohol analogue treatment.

To evaluate the cytotoxicity of the isolated compounds (coniferyl alcohol, coniferin, and *p*-coumaryl alcohol) and the coniferyl alcohol-related compound (sinapyl alcohol), we assessed their effects on SZ95 sebocyte viability (Figure 2). Remarkably, none of the tested compounds exhibited cytotoxic effects, and more than 80% of the SZ95 cells remained viable after treatment for 24 h with 100, 200, and 300 µM of these compounds.

### 2.3. Induction of Lipogenesis by Isolated Coniferyl Alcohol in LA-Stimulated SZ95 Sebocytes

**Figure 3:**
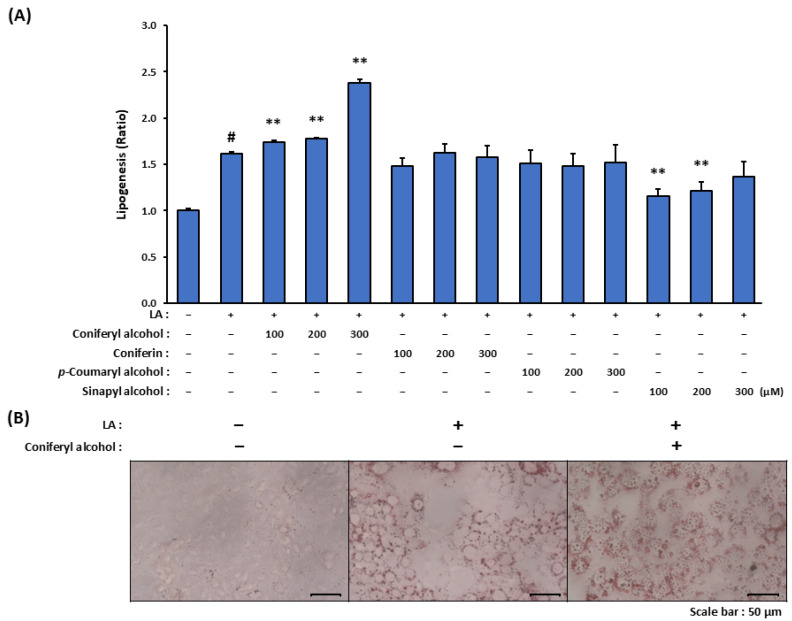
The effect of coniferyl alcohol and other cinnamyl alcohol analogues isolated from seed watermelon fruit on lipogenesis in LA-stimulated SZ95 sebocytes. (**A**) SZ95 cells were pre-treated with cinnamyl alcohol analogues (100, 200, and 300 µM; coniferyl alcohol, coniferin, *p*-coumaryl alcohol, and sinapyl alcohol) for 2 h. The cells were then stimulated with and without LA (100 µM) for 24 h. Following incubation, the cells were stained with Oil Red O, and the dye dissolved in the lipid droplets was extracted and measured using a colorimetric microplate reader. The data are presented as means ± standard error (*n* = 12) and were analyzed for statistically significant differences using Student’s *t*-test, with different letters indicating the differences at # *p* < 0.05, vs. SZ95 cells without both LA-stimulation and cinnamyl alcohol analogue treatment, ** *p* < 0.01 vs. LA-stimulated SZ95 cells with no cinnamyl alcohol analogue treatment. (**B**) SZ95 cells were pre-treated with coniferyl alcohol (300 µM) for 2 h. The cells were then stimulated with and without LA (100 µM) for 24 h. Following incubation, the cells were stained with Oil Red O and observed using a microscope.

LA strongly induces in vitro differentiation and lipid metabolism in sebocytes; specifically, it upregulates the genes involved in fatty acid metabolism, which leads to an increase in the intracellular levels of neutral lipids and thereby an enlargement in the associated lipid droplets. As detailed in this sub-section, we investigated whether the isolated compounds (coniferyl alcohol, coniferin, and *p*-coumaryl alcohol) and coniferyl alcohol-related compound (sinapyl alcohol) promoted lipogenesis in LA-stimulated SZ95 cells. Treatment with 100 µM LA alone resulted in a 1.6-fold increase in lipogenesis in the SZ95 cells (Figure 3A). Among the tested compounds, coniferyl alcohol significantly enhanced LA-induced lipogenesis in a dose-dependent manner, with 300 µM coniferyl alcohol leading to an approximately 2.2-fold increase in lipogenesis relative to the case of LA stimulation alone. These findings were further supported by microscopic analyses employing Oil Red O staining, which revealed enlarged lipid droplets in the LA-stimulated SZ95 cells treated with coniferyl alcohol (Figure 3B). Contrastingly, coniferin and *p*-coumaryl alcohol did not significantly influence the lipid synthesis. Sinapyl alcohol exhibited inhibitory effects on lipogenesis at concentrations of 100 and 200 µM; however, these effects were not observed at a concentration of 300 µM.

### 2.4. Influence of Isolated Coniferyl Alcohol on Lipid Profile of LA-Stimulated SZ95 Sebocytes

**Figure 4:**
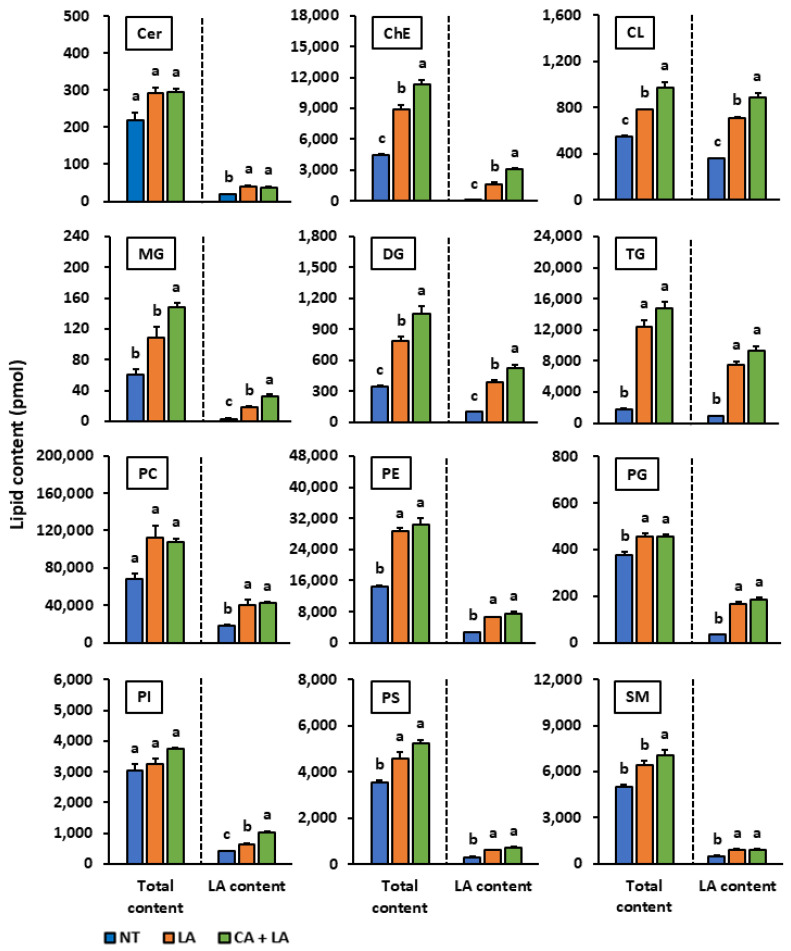
The effect of coniferyl alcohol isolated from watermelon fruit on the lipid profile in LA-stimulated SZ95 sebocytes. SZ95 cells were first pre-treated with coniferyl alcohol (300 µM) for 2 h and then stimulated with and without LA (100 µM) for 24 h. Following incubation, lipids were extracted from the cells using the Bligh and Dyer method. The 12 lipid profile classes [ceramide (Cer), cholesterol ester (ChE), cardiolipin (CL), monoglyceride (MG), diglyceride (DG), triglyceride (TG), phosphatidylcholine (PC), phosphatidylethanolamine (PE), phosphatidylglycerol (PG), phosphatidylinositol (PI), phosphatidylserine (PS), and sphingomyelin (SM)] were analyzed using liquid chromatography-electrospray ionization tandem mass spectrometry (LC-ESI/MS/MS). Total content: the total content of all lipid species including those containing various fatty acid components such as palmitic acid, stearic acid, oleic acid, and LA; LA content: content of lipid species that contain LA as one of their fatty acid components. The data are presented as means ± standard error (*n* = 3) and were analyzed for statistically significant differences using the Tukey–Kramer method, with different alphabets indicating the differences at *p* < 0.05. NT: SZ95 cells without both LA-stimulation and coniferyl alcohol treatment, LA: LA-stimulated SZ95 cells with no coniferyl alcohol treatment, CA + LA: LA-stimulated SZ95 cells with 300 µM coniferyl alcohol treatment.

We performed lipidomic analyses to investigate whether coniferyl alcohol alters the lipid composition and promotes lipid synthesis in LA-stimulated SZ95 sebocytes. We observed that LA stimulation significantly increased the cellular levels of various lipid species including cholesterol ester, cardiolipin, diglyceride, triglyceride, phosphatidylethanolamine, phosphatidylglycerol, and phosphatidylserine (Figure 4). Additionally, coniferyl alcohol treatment enhanced the accumulation of various lipid species including cholesterol ester, cardiolipin, simple lipid, and sphingomyelin. When comparing the contents of lipid species with and without LA, we found that the levels of LA-containing cardiolipin, phosphatidylglycerol, and phosphatidylinositol increased after coniferyl alcohol pretreatment and LA stimulation. Contrastingly, the levels of their non-LA-containing counterparts decreased or remained unchanged.

## 3. Discussion

In humans, sebum synthesis and secretion peak during adolescence and decline with age thereafter, resulting in approximately one in three elderly individuals experiencing the condition of dry skin [13,14]. Sebum lubricates the skin to protect against friction and makes it more impervious to moisture. Furthermore, the sebaceous gland transports antioxidants in and on the skin and exhibits a natural light protective activity [31]. It possesses an innate antibacterial activity and has a pro- and anti-inflammatory function [32]. It can regulate the activity of xenobiotics and is actively involved in the wound healing process [33]. In this study, we searched for natural compounds present in seed watermelon fruits that can help fundamentally improve sebum production. The active third fraction, obtained via solid-phase extraction using 20% CH_3_OH, contained several compounds including coniferyl alcohol, coniferin, and *p*-coumaryl alcohol (Figure 1). These compounds, classified as cinnamyl alcohol analogues, are precursors of lignin, a major plant cell wall component synthesized from the amino acids phenylalanine and tyrosine. Coniferyl alcohol is poorly water-soluble and possesses a free phenolic hydroxyl group with a high oxidative potential. In fresh shoots of *Ginkgo biloba* L. and *Magnolia liliiflora*, this compound undergoes the glycosylation of its phenolic hydroxyl group to give rise to coniferin, which results in increased water solubility and reduced oxidative activity [34]. This modification also facilitates the transport of coniferyl alcohol to the sites of lignin deposition [35].

We herein examined whether lipogenesis in LA-stimulated SZ95 sebocytes was promoted by the coniferyl alcohol, coniferin, *p*-coumaryl alcohol, and coniferyl alcohol-related compound sinapyl alcohol. According to our observations, coniferyl alcohol enhanced LA-induced lipogenesis in a dose-dependent manner (Figure 3A). Contrastingly, coniferin and *p*-coumaryl alcohol did not significantly influence lipid synthesis. The lack of activity observed for coniferin is presumably because of the glycosylation of its phenolic hydroxyl group, which likely reduced membrane permeability and cellular uptake [36,37]. Furthermore, we hypothesized that the presence of a methoxy group adjacent to the phenolic hydroxyl group enhanced lipid synthesis in the LA-stimulated SZ95 cells.

We subsequently conducted lipidomic analyses to elucidate these effects further and assess the influence of LA stimulation and coniferyl alcohol pretreatment on sebum synthesis. The total lipid content increased under LA stimulation, and coniferyl alcohol pretreatment further amplified this increase (Figure 4), which was consistent with the observations obtained upon Oil Red O staining. LA functions as a ligand for G protein-coupled receptor 120 (GPR120), which is expressed in various tissues including the small intestine, spleen, adipose tissue, and taste buds [38,39]. When LA binds to GPR120 in 3T3L1 cells, adipogenesis is triggered via the activation of peroxisome proliferator-activated receptor γ and upregulation of key adipogenic genes through intracellular calcium signalling and the extracellular signal-regulated kinase 1/2 signal pathway [40]. LA is also taken up by fatty acid transporters, serving as both an energy source and a major structural component of lipid membranes [41,42]. Once internalized, LA is rapidly converted to fatty acyl-CoA by acyl-CoA synthetase [43,44]. While some quantity of the fatty acyl-CoA is utilized for mitochondrial energy production, the remaining is stored in the form of lipid droplets. Our study showed that the levels of non-LA-containing cardiolipin, phosphatidylglycerol, and phosphatidylinositol either decreased or remained unchanged following LA stimulation and coniferyl alcohol pre-treatment, whereas the levels of LA-containing compounds increased (Figure 4). These results suggest that LA was selectively taken up and incorporated into specific lipid species in the SZ95 sebocytes, thereby directly contributing to lipid synthesis. We intend to continue investigating the mechanisms governing LA-induced lipogenesis in SZ95 sebocytes as well as the lipogenesis-promoting effects of coniferyl alcohol under LA stimulation.

Overall, our studies demonstrated that coniferyl alcohol isolated from the seed watermelon fruit promoted lipogenesis in LA-stimulated SZ95 sebocytes. These findings suggest that seed watermelon can serve as a raw ingredient in the development of newer skin topical applications and that remedies containing seed watermelon fruit extracts in combination with LA may benefit individuals with dry skin. These identified active compounds are lignin precursors, which is significant as lignin is a component of the seed coat. Related studies have shown that pomegranate cultivars possessing hard seeds accumulated higher levels of coniferyl alcohol and sinapyl alcohol in the inner seed coat than soft-seeded varieties did [45]. The seed size and seed coat thickness in watermelons vary depending on the cultivated variant, with seed watermelons possessing a relatively thicker seed coat than that of other variants [46,47]. If watermelons exhibit a trend similar to that of pomegranates, varietal differences in lignin precursor contents can be expected. Therefore, we aim to continue screening and selecting watermelon varieties to identify those with the optimal potential to promote sebum synthesis.

## 4. Materials and Methods

### 4.1. Materials

Coniferin was purchased from MedChemExpress (Monmouth Junction, NJ, USA). Coniferyl alcohol was purchased from FUJIFILM Wako Pure Chemical Corporation (Osaka, Osaka, Japan). Sinapyl alcohol was purchased from Cayman Chemical (Ann Arbor, MI, USA).

### 4.2. Isolation

Whole seed watermelon fruits were crushed, pressed, and concentrated to obtain the seed watermelon fruit extract. The crude extract was dissolved in a small volume of H_2_O, applied to an InertSep C18 column (10 g), and eluted stepwise with the following solvents: 100% H_2_O; 9:1, 4:1, 7:3, 3:2, and 1:1 (*v*/*v*) H_2_O–CH_3_OH; and 100% CH_3_OH (60 mL each). The third fraction obtained in 4:1 H_2_O–CH_3_OH was further purified via reverse-phase HPLC employing a C-18 packed column (10 mm × 250 mm; PEGASIL ODS sp100, Senshu Scientific Co., Tokyo, Japan) under the following conditions: 20–40% aq. CH_3_OH used as solvent, linear gradient of 30 min; flow rate of 3.0 mL/min, and detection using ultraviolet (UV) light at a wavelength of 254 nm.

### 4.3. Cell Culture

The immortalized human sebaceous gland cell line SZ95 was obtained from Professor Christos C. Zouboulis (Staedtisches Klinikum Dessau, Dessau, Germany) [48]. The cells were cultured in EpiLife^TM^ medium supplemented with 60 µM calcium (Thermo Fisher Scientific, Waltham, MA, USA), 10% heat-inactivated fetal bovine serum (CORNING, Corning, NY, USA), penicillin (50 U/mL), streptomycin (50 µg/mL), and recombinant human epidermal growth factor (0.2 µg/mL, Thermo Fisher Scientific) in a humidified atmosphere containing 5% CO_2_ at 37 °C.

### 4.4. Cell Viability Assay

The SZ95 cells were seeded into 24-well plates (2 × 10^5^ cells/well) and cultured for 48 h. After 48 h of incubation, the cells were treated with coniferyl alcohol, coniferin, *p*-coumaryl alcohol, and sinapyl alcohol for 24 h at final concentrations of 100, 200, and 300 µM. Subsequently, the culture medium was replaced with fresh medium containing 10% WST-1 (Premix WST-1 Cell Proliferation Assay System, Takara Bio Inc., Ohtsu, Shiga, Japan). After incubation at 37 °C for 1 h, the absorbance was measured at wavelengths of 450 and 690 nm using a colorimetric microplate reader (Varioskan LUX, Thermo Fisher Scientific). The percentage of viable cells was calculated using Equation (1) as follows.

(1) $$\begin{array}{c} Cell\;viability\left(\%\right)\\ \;\;\;=\frac{\left({Abs}_{450nm}-{Abs}_{690nm}\right)\;of\;cells\;treated\;with\;Sample}{\left({Abs}_{450nm}-{Abs}_{690nm}\right)\;of\;cells\;treated\;without\;sample}\times 100 \end{array}$$

### 4.5. Oil Red O Staining

The SZ95 cells were seeded into 24-well plates (2 × 10^5^ cells/well) and cultured for 48 h. After 48 h of incubation, the cells were pre-treated with coniferyl alcohol, coniferin, *p*-coumaryl alcohol, and sinapyl alcohol for 2 h at final concentrations of 100, 200, and 300 µM. Subsequently, the cells were stimulated with and without LA at a final concentration of 100 µM (Sigma-Aldrich, St. Louis, MO, USA) for 24 h. After 24 h of incubation, the culture medium was replaced with fresh medium containing 10% WST-1 (Premix WST-1 Cell Proliferation Assay System, Takara Bio Inc.). After incubation at 37 °C for 1 h, the absorbance was measured at wavelengths of 450 and 690 nm using a colorimetric microplate reader (Varioskan LUX, Thermo Fisher Scientific). The cells were then washed twice using phosphate-buffered saline (PBS)(-) and fixed in 10% formalin at 4 °C overnight. The fixed cells were also washed twice using PBS(-) and stained for 1 h using a staining solution composed of Oil Red O dye (Cosmo Bio Co., Ltd., Tokyo, Japan) and ultrapure H_2_O in a *v*/*v* ratio of 3:2. After the staining process, the cells were washed using distilled H_2_O and dried at 25 °C for 1 h. The cells stained with Oil Red O were observed under a microscope (BZ-X800, Keyence Co., Ltd., Osaka, Japan) with a 40× objective lens. The Oil Red O content dissolved in the lipid droplets was extracted using 2-propanol, after which the absorbance was measured at a wavelength of 540 nm using a colorimetric microplate reader (Varioskan LUX, Thermo Fisher Scientific). The lipogenesis (ratio) was calculated using Equation (2) as follows.

(2) $$\begin{array}{c} \;\;Lipogenesis\left(ratio\right)\\ \;\;=\frac{{Abs}_{540nm}-\left({Abs}_{450nm}-{Abs}_{690nm}\right)\;of\;cells\;treated\;with\;Sample}{{Abs}_{540nm}-\left({Abs}_{450nm}-{Abs}_{690nm}\right)\;of\;cells\;treated\;without\;Sample} \end{array}$$

### 4.6. Non-Targeted Lipidomics Analysis (LC-ESI/MS/MS Analysis)

#### 4.6.1. Lipid Preparation

Methanol, isopropanol, and chloroform of ultra-performance LC/MS quality were obtained from FUJIFILM Wako Pure Chemical Corporation (Osaka, Japan). Ultrapure water was obtained from a Milli-Q water system (Millipore, Milford, MA, USA). Lipidome analysis was conducted using the Lipidome lab Non-targeted Lipidome Scan package (Lipidome lab, Akita, Japan) via LC orbitrap MS, as per methods described previously [49,50]. As part of the extraction of lipid metabolites from the SZ95 sebocytes, the cells were seeded in 6-well plates at a density of 2 × 10^5^ cells/mL and treated with and without coniferyl alcohol at a final concentration of 300 µM following LA stimulation for 24 h. The cells were then washed with PBS(-), harvested, dissolved with methanol, and homogenized. The total lipid content in this solution was extracted using the Bligh and Dyer liquid–liquid extraction methods. An aliquot of the lower/organic phase was evaporated to dryness under nitrogen gas, and the residue was re-dissolved in methanol for LC-MS/MS measurements.

#### 4.6.2. Instrumental Analysis and Data Processing

All internal standard reagents were purchased from Avanti Polar Lipids (Alabaster, AL, USA). LC-ESI/MS/MS was performed using a Q-Exactive Plus mass spectrometer with an UltiMate 3000 LC system (Thermo Fisher Scientific). Samples were separated on an L-column3 C18 metal-free column (2.0 µm, 2.0 mm × 100 mm i.d.) at 40 °C using a gradient solvent system. Mobile phase A comprised a mixture of isopropanol, methanol, and water (5/1/4 *v*/*v*/*v*) supplemented with 5 mM ammonium formate and 0.05% ammonium hydroxide (28% in water). Mobile phase B comprised isopropanol supplemented with 5 mM ammonium formate and 0.05% ammonium hydroxide (28% in water). The gradient flow consisted of the following ratios: 60% A/40% B (0 min), 40% A/60% B (0–1 min), 20% A/80% B (1–9 min), 5% A/95% B (9–11 min), 5% A/95% B (11–22 min), 95% A/5% B (22–22.1 min), 95% A/5% B (22.1–25 min), 60% A/40% B (25–25.1 min), and 60% A/40% B (25.1–30 min). The injection volume was 10 µL, and the flow rate was 0.1 mL/min. Conditions for the heated electrospray ionization source were as follows: ionization mode, positive or negative; sheath gas, 60 arbitrary units; auxiliary gas, 10 arbitrary units; sweep gas, 0 arbitrary units; spray voltage, 3.2 kV in positive mode and −3.0 kV in negative mode; heater temperature, 325 °C; ion transfer capillary temperature, 300 °C in positive mode and −320 °C in negative mode; and S-lens RF level, 50. The Orbitrap mass analyzer was operated at a resolving power of 70,000 in full-scan mode (scan range: 200–1800 *m/z* in both positive and negative modes; automatic gain control (AGC) target of 1e6 in positive mode and 3e6 in negative mode) and a resolving power of 17,500 in positive mode and 35,000 in negative mode in the Top 20 data-dependent MS2 mode (stepped normalized collision energy: 20, 30, and 40; isolation window: 4.0 *m/z*; AGC target: 1e5) with a dynamic exclusion setting of 10.0 s.

Post-processing of the raw data files was performed using the lipid molecular identification software, Lipid Search 5.1 (Mitsui Knowledge Industries Co., Ltd., Tokyo, Japan). This software identifies individual intact lipid molecules based on their molecular weight, fragmentation patterns, and headgroup and fatty acid compositions. In this method, biological matrix effects cannot be normalized across all detected peaks, because it is not possible to prepare appropriate internal standards for all the detected peaks. The relative values were calculated using the ratios of the chromatographic peak areas of each analyte to that of the total analyte peak. However, for some lipid classes, values were calculated by normalizing them using internal standards. The quantification and annotation methods used in this study correspond to the “absolute quantification Level 2 or 4” and “Fatty Acyl/Alkyl Level or Hydroxyl Group Level” defined by the Lipidomics Standard initiative, respectively [51].

### 4.7. Statistical Analysis

All data were analyzed using the Mac statistical analysis software package for Macintosh (version 2.0; Esumi Co., Tokyo, Japan). All data and results are expressed as means ± standard error. Statistical significance was assessed using the Tukey–Kramer test or Student’s *t*-test, with * *p* values < 0.05 and ** *p* values < 0.01 considered as statistically significant.

## Data Availability

All data are contained within the article and Supplementary Materials.

## Supplementary Materials

The following supporting information can be downloaded at: https://www.mdpi.com/article/10.3390/molecules30163360/s1, Figure S1: The effect of each fraction separated from seed watermelon fruit on lipogenesis in LA-stimulated SZ95 sebocytes, Figure S2: The effect of each fraction separated from the third Fr. on lipogenesis in LA-stimulated SZ95 sebocytes.

## Abbreviations

The following abbreviations are used in this manuscript:

UV	Ultraviolet
DHT	5α-dihydrotestosterone
LA	Linoleic acid
HPLC	High-performance liquid chromatography
CA	Coniferyl alcohol
Cer	Ceramide
ChE	Cholesterol ester
CL	Cardiolipin
MG	Monoglyceride
DG	Diglyceride
TG	Triglyceride
PC	Phosphatidylcholine
PE	Phosphatidylethanolamine
PG	Phosphatidylglycerol
PI	Phosphatidylinositol
PS	Phosphatidylserine
SM	Sphingomyelin
LC-ESI/MS/MS	Liquid chromatography-electrospray ionization tandem mass spectrometry
GPR120	G protein-coupled receptor 120
PBS	Phosphate-buffered saline
AGC	Automatic gain control
