# Supplementary material for: Seed Watermelon (Citrullus mucosospermus (Fursa))-Derived Coniferyl Alcohol as a Functional Ingredient in Remedies for Dry Skin: Evidence of Facilitated Lipogenesis in Human Sebocytes
Source: Molecules. 2025 Aug 13;30(16):3360. doi: 10.3390/molecules30163360 (PMC12388719; doi:10.3390/molecules30163360)
Supplement: Supplementary file 1 [file molecules-30-03360-s001.zip › molecules-3754516-supplementary.pdf]

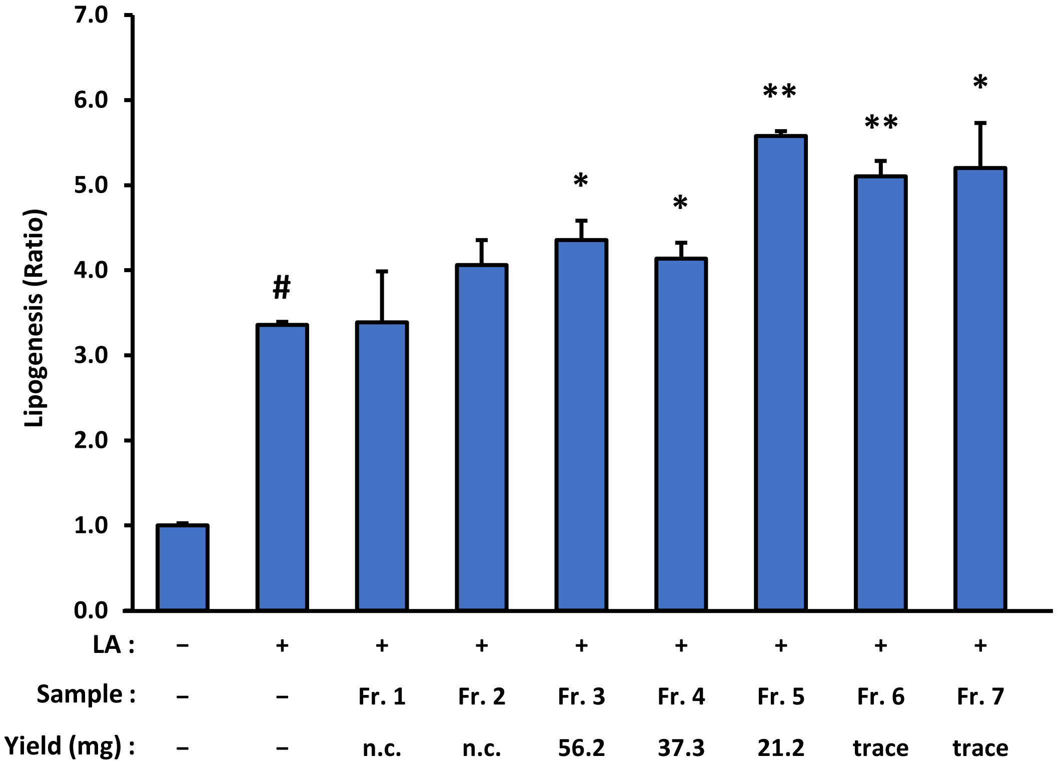

Figure. S1 The effect of each fraction separated from seed watermelon fruit on lipogenesis in LA-stimulated SZ95 sebocytes. SZ95 cells were pre-treated with each fraction (100% H_2O (Fr. 1); 9:1 (Fr. 2), 4:1 (Fr. 3), 7:3 (Fr. 4), 3:2 (Fr. 5), and 1:1 (Fr. 6) (v/v) H_2O–CH_3OH; and 100% CH_3OH (Fr. 7)) for 2 h. The cells were then stimulated with and without LA (100 μM) for 24 h. Following incubation, the cells were stained with Oil Red O, and the dye dissolved in the lipid droplets was extracted and measured using a colorimetric microplate reader. The data are presented as means ± standard error (n = 9) and were analyzed for statistically significant differences using Student's t-test, with different letters indicating the differences at #$p < 0.05$, *vs* SZ95 cells without both LA-stimulation and sample treatment, *$p <$ 0.1 and **$p < 0.01$ *vs* LA-stimulated SZ95 cells with no sample treatment. n.c.: not calculated.

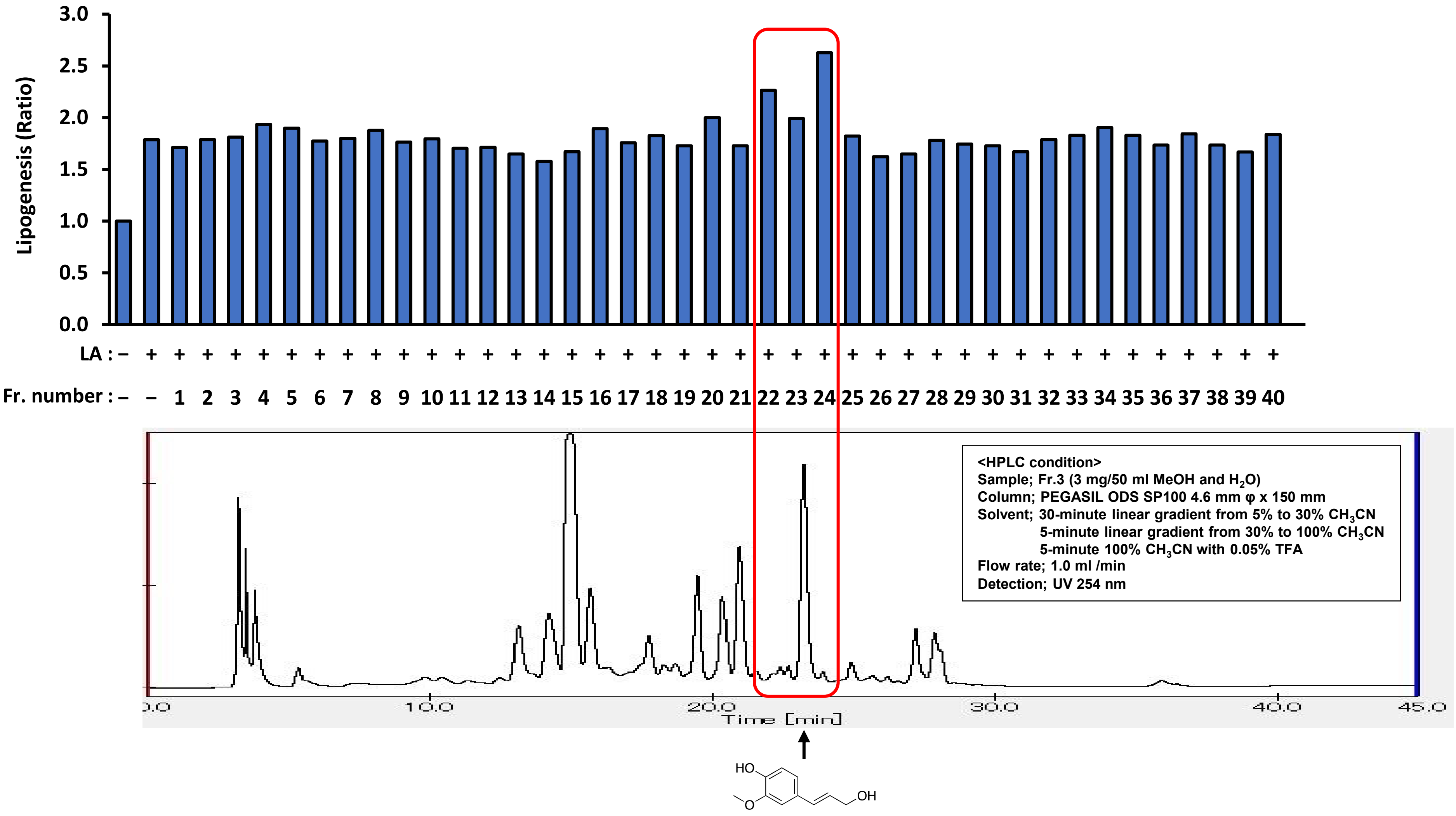

Figure. S2 The effect of each fraction separated from the third fraction on lipogenesis in LA-stimulated SZ95 sebocytes. Each fraction was collected at one-minute intervals separated from the third fraction, under the HPLC conditions shown in the figure. The fraction numbers correspond to the retention times in the HPLC analysis. SZ95 cells were pre-treated with each Fr. for 2 h. The cells were then stimulated with and without LA (100 μM) for 24 h. Following incubation, the cells were stained with Oil Red O, and the dye dissolved in the lipid droplets was extracted and measured using a colorimetric microplate reader.
